# Multiple dimensions of biodiversity drive human interest in tide pool communities

**DOI:** 10.1038/s41598-018-33155-x

**Published:** 2018-10-15

**Authors:** Tom P. Fairchild, Mike S. Fowler, Sabine Pahl, John N. Griffin

**Affiliations:** 1 Department of Biosciences, Swansea University, Singleton Park Campus, Swansea, SA2 8PP United Kingdom; 2 School of Psychology, University of Plymouth, 22 Portland Square, Drake Circus, Plymouth, Devon PL4 8AA United Kingdom

## Abstract

Activities involving observation of wild organisms (e.g. wildlife watching, tidepooling) can provide recreational and learning opportunities, with biologically diverse animal assemblages expected to be more stimulating to humans. In turn, more diverse communities may enhance human interest and facilitate provisioning of cultural services. However, no experimental tests of this biodiversity-interest hypothesis exist to date. We therefore investigated the effects of different dimensions of animal biodiversity (species richness, phyletic richness and functional diversity) on self-reported interest using tide pools as a model system. We performed two experiments by manipulating: (1) the richness of lower (species) and higher taxonomic levels (phyla) in an image based, online survey, and (2) the richness of the higher taxonomic level (phyla) in live public exhibits. In both experiments, we further quantified functional diversity, which varied freely, and within the online experiment we also included the hue diversity and colourfulness arising from the combination of organisms and the background scenes. Interest was increased by phyletic richness (both studies), animal species richness (online study) and functional diversity (online study). A structural equation model revealed that functional diversity and colourfulness (of the whole scene) also partially mediated the effects of phyletic richness on interest in the online study. In both studies, the presence of three of four phyla additively increased interest, supporting the importance of multiple, diverse phyla rather than a single particularly interesting phylum. These results provide novel experimental evidence that multiple dimensions of biodiversity enhance human interest and suggest that conservation initiatives that maintain or restore biodiversity will help stimulate interest in ecosystems, facilitating educational and recreational benefits.

## Introduction

Ecosystems underpin human wellbeing through their provisioning, regulating, supporting and cultural services^1–3^. Cultural services include ecosystems’ ability to stimulate educational benefits^4,5^, relieve stress^1,6,7^, revitalize the brain’s ability to direct attention^6,8,9^ and provide enjoyment through recreation^10^. Yet, despite a growing understanding of the general importance of ecosystems for cultural services^6,11,12^, experimental studies examining the specific role of biodiversity (i.e., the variety of life, including diversity within and between species^3^) in facilitating these services are rare. However, explicitly considering links between biodiversity and facilitation of cultural services may inform management of ecosystems to enhance the provisioning of services^12,13^, support public engagement (e.g. aquarium exhibits^14^ or ecotourism opportunities) and promote biodiversity conservation^15^.

Humans choose to engage with nature through activities such as wild food foraging and wildlife watching, tapping a flow of recreational and educational services^16^ which depend on organisms present within ecosystems. Ecosystems with greater numbers of taxa both tend to incorporate a broader range of organismal traits (functional diversity)^17^, many of which are visible and conspicuous to the human observer (e.g. organism colour, body shape, locomotion), and are more likely to include taxa with traits towards the extremes of trait-space^18^ (e.g. large size, bright colours). These attributes of biodiverse ecosystems coincide strongly with empirical findings within the psychology of interest: novelty, complexity and vividness increase human interest, which, in turn, triggers exploration, intrinsic motivation and learning^19^. Thus, the emotion of interest is functional and drives action choice. Particularly, novelty in objects, animals or scenes can drive interest by providing unusual stimuli, and can operate independently of aesthetic pleasure and preference^20–22^, with subjects often appearing interesting even where they are not aesthetically pleasing^22,23^. While there has been some ambiguity in the definition of “interest”^20,24–26^, here we use a definition from psychological science, as: “the need to give selective attention to something which has significance to a person”^20^. In turn, this emotion of interest can play a crucial, functional role, determining human action selection^27^ and development of knowledge and competence^19,20,24^. Ecosystems with greater taxonomic diversity (e.g., more species, families and/or phyla) and functional diversity (greater diversity in functional traits that define organisms) may therefore enhance human interest and provide pathways to cultural services by improving educational and aesthetic experience opportunities. Yet, despite compelling links between these ecological and psychological perspectives, and some studies examining the effects of different aspects of biodiversity on cultural components such as aesthetic preference^28–30^ and wellbeing^7,31,32^, no explicit experimental tests of the connection between biodiversity dimensions and the functional emotion of interest exist to date.

Therefore, we experimentally investigated the role of animal biodiversity in influencing human interest in ecosystems using tide pools as a model. Tidepooling (also known as rockpooling), observing and collecting marine organisms in tide pools, is popular on rocky shores worldwide^33–35^. Tide pools provide a window into marine ecosystems, allowing people to observe and interact with animals, bringing recreational and educational benefits^33,34,36,37^. These pools are also often naturally biodiverse, with many co-occurring species, functional forms and phyla^38^. Tidepools also form an analogue to many other accessible ecosystems, such as grasslands, woodlands and uplands, containing diverse communities of smaller organisms which are often not defined by the presence of charismatic species^39,40^, but are easily accessed and frequented by visitors^41^.

We conducted two complementary experiments to understand how different levels of biodiversity (i.e. species and phyletic richness, and functional diversity) influence human interest as a facilitator of cultural ecosystem services. We incorporated phyletic richness in addition to species richness and functional diversity, as traits tend to diverge with evolutionary and thus taxonomic distance^42^. As such, richness at higher taxonomic levels (e.g. phylum) may capture important additional aspects of traits (e.g. variation in body plan), and accordingly be easier for lay observers to differentiate. Because animals vary in their body colours, and human preferences for scenes have been shown to respond strongly to colour characteristics^25^, we also explicitly quantified both the average colourfulness (vividness) and the diversity of colour hues within scenes as a whole (capturing the interplay of contained organisms, and background tidepool scenes), to better understand the scene properties and potential mechanisms linking biodiversity and interest.

The first experiment was an image-based online study to understand how biodiversity affects self-reported interest in a tide pool image. To further understand these effects, we also explored whether and how the influence of species and phyletic richness on interest was mediated by functional diversity (indicating trait complementarity), and the scene-level properties of hue diversity and colourfulness. The second experiment used live animals in simulated tide pools at a public exhibit to examine whether biodiversity effects hold under more natural settings, focusing on the role of biodiversity at a higher taxonomic (phylum), and functional level. We investigated the following hypotheses: (1) increasing richness of both lower (species) and higher (phylum) taxonomic levels, and of functional diversity, all increase interest; (2) the effects of taxonomic diversity are partially mediated by functional diversity and the scene properties of colour hue diversity, and/or colourfulness; and (3) multiple phyla, rather than any single phylum alone, increase interest.

## Results

### Image based online study

Of the 741 people that responded to the online study, 601 completed the image-based questions, and 527 people completed the study and demographics in full, giving a completion rate of 71.1%. Demographic background did not influence interest in images (Supplementary Table 1).

**Figure 1:**
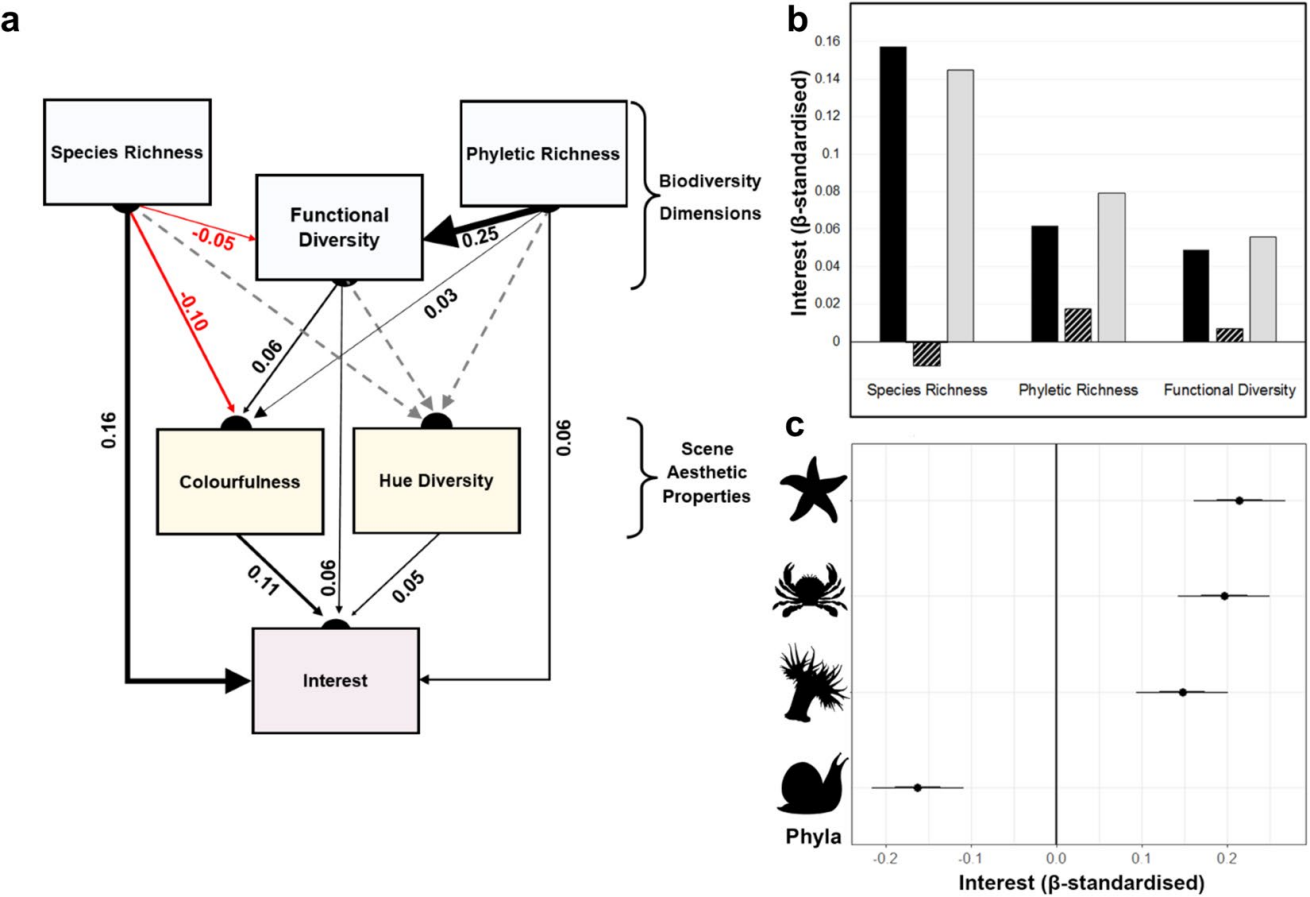
The effects of diversity and aesthetic components on interest in the online study, with: (**a**) Piecewise SEM path model of taxonomic and aesthetic components on interest for the online study. Black lines indicate significant positive effects, red indicate significant negative effects and dashed lines indicate relationships that were non-significant; (**b**) Direct (black), indirect (hatched) and net (light grey) effects of diversity components on interest; and (**c**) Coefficient plots of the effects of phyletic identity on interest, for (from top to bottom) Echinodermata, Arthropoda, Cnidaria and Mollusca. Inner bars represent a 1-standard deviation CI (68%), and outer bars a 2-standard deviation CI (95%). All regression estimate effect sizes are standardised.

The presence of animals in images increased interest significantly (β = 1.893, S.E. = 0.056, Z = 41.2, p < 0.0001), from a mean interest value of 0.30 (±0.21 S.D.) where no animals were included, to 0.66 (±0.22 S.D.) where animals were present. There was also no systematic preference for base tidepool scenes (ChiSQ = 2.471, *Df* = 2, p = 0.291). Interest in images varied considerably among participants (Random effects variance = 0.8929), but generally increased with all biodiversity and colour components. Increasing species and phyletic richness, and functional divergence (our chosen metric of functional diversity, see Methods) all increased average reported interest. Notably, however, the positive (standardised) direct effect of species richness (β = 0.158 ± 0.013 S.E. p < 0.001; Table 1, Fig. 1a) was stronger than that of either phyletic richness (β = 0.062 ± 0.015 S.E., p = 0.001) or functional diversity (β = 0.049 ± 0.015 S.E. p = 0.001). The scene-level properties of colourfulness and hue diversity also both increased interest, with colourfulness having the larger effect (colourfulness: β = 0.109 ± 0.015 S.E., p < 0.001; hue diversity: = 0.051 ± 0.015 S.E., p < 0.001, Table 1, Fig. 1a), although this effect was still weaker than that of species richness.

**Table 1:** The effects of taxonomic richness, functional diversity and aesthetic components on interest, and SEM sub models for the online study (SEM model 1). Estimates are standardised β-regression coefficients.

Response	Predictor	Estimate (β)	SE	P value
Functional Diversity	Phyletic Richness	0.252	0.008	<0.001
Functional Diversity	Species Richness	−0.045	0.007	<0.001
Colourfulness	Species Richness	−0.095	0.010	<0.001
Colourfulness	Functional Diversity	0.062	0.012	<0.001
Colourfulness	Phyletic Richness	0.031	0.012	0.010
Hue Diversity	Functional Diversity	−0.002	0.002	0.354
Hue Diversity	Phyletic Richness	0.002	0.002	0.380
Hue Diversity	Species Richness	−0.001	0.002	0.520
Interest	Species Richness	0.158	0.013	<0.001
Interest	Colourfulness	0.109	0.015	<0.001
Interest	Phyletic Richness	0.062	0.015	<0.001
Interest	Hue Diversity	0.051	0.015	<0.001
Interest	Functional Diversity	0.049	0.015	0.001

The structural equation model (SEM) also revealed indirect effects of dimensions of biodiversity. Surprisingly, species richness weakly reduced functional diversity and colourfulness, so these variables transmitted a negative indirect effect between species richness and interest. This slightly tempered the positive net effect of species richness (Fig. 1b). Since phyletic richness increased functional diversity and colourfulness, these variables transmitted positive indirect effects of phyletic richness on to interest, strengthening its net effect. Even after accounting for opposing indirect effects, the net effect of species richness still exceeded that of phyletic richness, and functional diversity (Fig. 1b). Hue diversity was not significantly influenced by either of the taxonomic measures or functional diversity, and did not mediate relationships between phyletic or species richness and interest (Table 1).

We also expected that phylum identity would affect interest in images (Fig. 1c). The presence of Echinodermata, Arthropoda and Cnidaria in images all increased interest, whereas Mollusca reduced interest (Table 2). Therefore, although phyla varied in their contributions to interest, three of four phyla had positive effects, indicating that the positive effect of phyla richness could not be attributed to a single particularly interesting phylum.

**Table 2:** The effects of phyletic identity on interest in the online study. Estimates are standardised β-regression coefficients.

Phyla	Estimate (β)	SE	Z-value	P value
Arthropoda	0.197	0.027	7.29	<0.0001
Cnidaria	0.147	0.027	5.48	<0.0001
Mollusca	−0.163	0.027	−6.07	<0.0001
Echinodermata	0.215	0.027	7.99	<0.0001

### Public exhibit study

114 people took part in the public exhibit study of which 97 provided demographic information. Neither age nor gender had any significant effect on interest (Supplementary Table 2).

**Figure 2:**
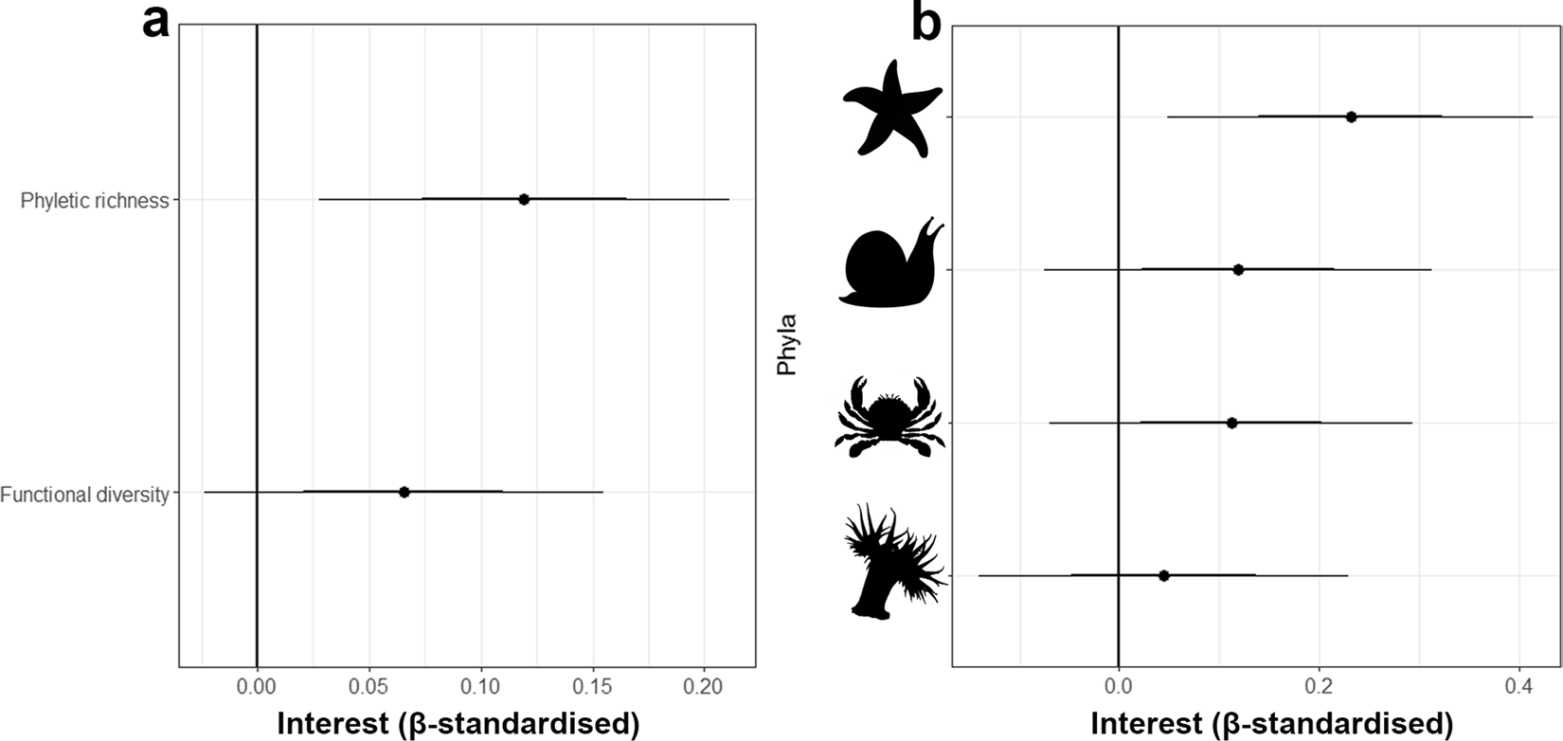
The effects of diversity and aesthetic components on interest in the public exhibit, with; (**a**) Coefficient plots of direct, partial effects of diversity components on interest, with inner bars representing a 1-standard deviation CI (68%), and outer bars a 2-standard deviation CI (95%); (**b**) Coefficient plots of partial effects of phyletic identity on interest in a scene for the public exhibit study for (from top to bottom) Echinodermata, Mollusca, Arthropoda and Cnidaria. Inner bars represent 1 standard deviation, and outer bars 2 standard deviations. All regression estimate effect sizes are standardised.

The presence of animals in tanks enhanced interest significantly (β = 1.325 SE = 0.125, Z = 10.61, p < 0.0001), increasing from a mean interest of 0.42 (±0.13 S.D.) where no animals were included, to 0.82 (±0.18 S.D.) where animals were present. Both phyletic richness and functional diversity also enhanced interest (Table 3). However, phyletic richness effects were not mediated through functional diversity, and there was no direct effect of functional diversity (Fig. 2a, Table 3). The presence of three of the four phyla (Echinodermata, Mollusca and Arthropoda) significantly increased interest (Fig 2b; Table 4).

**Table 3:** The effects of phyletic richness and functional diversity on interest and SEM sub models for in the public exhibit study (SEM model 2). Estimates are standardised β-regression coefficients.

Response	Predictor	Estimate (β)	SE	P value
Functional Diversity	Phyletic Richness	0.038	0.024	0.122
Interest	Phyletic Richness	0.149	0.046	0.001
Interest	Functional Diversity	0.119	0.045	0.008

**Table 4:** The effects of phyletic identity on interest in the public exhibit study. Estimates are standardised β-regression coefficients.

Phyla	Estimate (β)	SE	Z-value	P value
Arthropoda	0.380	0.089	4.29	<0.001
Cnidaria	0.151	0.093	1.63	0.100
Mollusca	0.413	0.093	4.46	<0.001
Echinodermata	0.382	0.089	4.28	<0.001

## Discussion

To summarise our key results, first, the diversity of animal species, phyla and functional traits drove human interest in tide pool communities (Fig. 1a). Second, aspects of biodiversity were linked not only to each other, but also to scene-level aesthetic properties, revealing both direct and indirect links between biodiversity and interest (Fig. 1a). Finally, positive effects of biodiversity on interest manifested in both image-based surveys, and in real-life contexts with live animals (Fig. 2a). Collectively, these results provide novel experimental evidence that multiple dimensions of biodiversity can drive human interest in ecological communities. Furthermore, Human interest is a functional emotion linked to action choice and thus greater interest is likely to lead to greater engagement with natural systems that, in turn, can facilitate the provision of cultural ecosystem services.

Of the three dimensions of biodiversity considered in our online study, species richness was the strongest driver of interest (Fig. 1a). There may be a number of non-exclusive explanations for this strong and exclusively direct positive effect. It is possible that people’s interest increased with a greater number of species expressing small functional differences in similar, desirable traits. This mechanism has been demonstrated for animal preference^43^. Indeed, functional diversity (divergence) slightly decreased with species richness (Fig. 1a), indicating clustering of species in a limited region of trait-space. On the other hand, viewers may have been responding to the volume of total trait space occupied (functional richness^30,44^), which is driven by species at the extremes of trait space and tends to strongly correlate with species richness^17,44^, but could not be applied in our study^45^ (see methods). A sampling effect may also be in operation, whereby particular species, including those that garner most interest from people, are more likely to be included as richness increases. However, this sampling effect was not the result of including colourful species; instead, species richness weakly suppressed colourfulness, likely through increasing the probability of including species with less vivid colours. Whatever the exact mechanisms and pathways, our results indicate that species richness is a key dimension of biodiversity driving human interest in ecosystems, a finding in agreement with previous studies which focused on the allied properties of human aesthetic preferences for scenes dominated by plants^28,29^, marine organisms^30^, and those which have examined diversity as a driver of attractiveness of wildlife watching activities^46,47^.

Although weaker than the effects of species richness, both functional diversity and phyletic richness additively contributed to interest in our online study. Independently of the number of species, people therefore seem to respond to differences among species, whether that is trait variation potentially occurring across phyla (e.g., body plan), or trait variation that we directly measured and incorporated into our metric of functional diversity. Tide pool scenes with high trait diversity are presumably visually and intellectually stimulating, since organisms with different body shapes, sizes, colours and textures all co-occur and juxtapose, providing complementary stimuli to the observer. Further, species with greater trait differences are presumably easier to distinguish, thus increasing the participants’ perceived diversity of communities^31^. As expected, phyletic and functional aspects of biodiversity were not independent, with the positive effect of phyletic richness on functional diversity indicating that phyla differed to some degree in measured traits and leading to an indirect effect of phyletic richness on interest. The other indirect effect of phyletic richness was via scene colourfulness, which is understood to enhance interest^19,22^ and aesthetic responses^48–50^. This probably reflects a sampling effect driven by the presence of colourful, warm hued organisms that contrast with the background^51^. Overall, results from our online study show that multiple dimensions of biodiversity influence interest and do so through different pathways illustrating that multiple and varied mechanisms likely link biodiversity and interest.

Image-based, online surveys provide experimental control and large sample sizes but miss animal movement and behaviour which can enhance human engagement with natural scenes^52,53^. It is notable, therefore, that we also found evidence to support a positive effect of biodiversity on interest within our smaller public exhibit study. This showed that phyletic richness effects on interest were maintained in a setting that allowed animal movement and behaviour, which can alter human interest in organisms^52,53^ (Fig. 2a). However, unlike in the online study, our SEM did not find evidence for mediation by functional diversity (Table 3), and nor did functional diversity have a statistically significant independent effect (P = 0.07). The loss of these pathways in the public exhibit may be due to lower variation in functional diversity in the four species assemblages presented, and/or lower replication. Nevertheless, the direct effect of phyletic richness suggests it captured more visibly obvious, and higher level, geometric, morphological and perhaps behavioural traits which were important in determining interest but missed from the functional diversity metric^54^.

Our analysis of the effects of individual phyla, for both the online and public exhibit studies, shows that three of four phyla enhanced interest in both studies, while none of these three phyla had a disproportionate effect on interest compared with the others (Tables 2 and 4). Coupled with the positive relationship between phyletic richness-interest (Fig. 1, Tables 1 and 3), this supports a role of phyletic richness in driving interest, rather than simply the chance inclusion of a particularly interesting phylum^50^. Furthermore, the sets of phyla that collectively enhanced interest differed between studies, hence across the two studies all phyla enhanced interest in tide pools^55^. The importance of phyletic richness is further supported by the mediation of its effect through functional diversity in the online study, which indicates a role of trait complementarity emanating from the combination of multiple phyla^56^.

Our finding that biodiversity enhances self-reported human interest in tide pool communities has potential implications in other marine and terrestrial settings. Natural coastal ecosystems around the world are under growing anthropogenic pressure^57–60^ and are increasingly being replaced through artificial hard coastal defence or renewable energy structures^61–63^ which themselves may host ecological communities^61,64,65^. These coastal areas are also popular destinations for tourism^10,66,67^ and recreation^10,35,68–70^ and provide learning opportunities^34,35,71^. Our study based on diverse marine animals suggests that managing and enhancing natural and human-made coastal habitats for biodiversity^72–75^ may increase public interest and thus subsequently enhance educational, recreational and tourism value, strengthening the case for managing coastal structures to improve biodiversity^76^. Furthermore, our findings hint that activities similar to tidepooling that provide valuable but declining wildlife experiences^77^, such as nature walks, bird watching, and fishing, may generate greater levels of human interest and engagement if a greater diversity of animals is present.

Our work may also find wider application; augmenting biodiversity in other habitats and learning centres may also enhance interest and value. For example, while public aquaria^78–80^, zoological museums^78,81–83^, and wildlife tourism activities^14,84^ are often designed and managed around charismatic species, promoting a greater variety of species, functional forms and higher taxonomic classifications may increase interest, visitor satisfaction, and ultimately educational value. Indeed, while there is growing evidence that rare, threatened or charismatic organisms can disproportionately influence interest and appreciation^14,78,85,86^, more diverse communities provide a greater variety of species which appeal to different people^27,46^. This may increase the value of biodiversity across different cross sections of the public, even where particularly charismatic species exist^46,47,87^.

However, it is important to acknowledge that while care was taken to represent a cross-section of demographic groups, recruitment of participants via social media (online study) and *in-situ* at a marine reserve may not capture the full spectrum of potential users^88^. Furthermore, explicit examination of the relative roles of biodiversity and individual species’ traits and behaviours, within and beyond tide pools, are needed to more fully understand the mechanisms that drive interest in natural systems.

In conclusion, we show here that multiple aspects of biodiversity determine human interest in tide pools, providing the first direct experimental link between the functional emotion of interest and biodiversity that is likely to facilitate the flow of recreational and educational benefits from ecosystems. There has been growing interest in cultural services, but researchers have only scratched the surface of the link between biodiversity and the delivery of such services. It is imperative that these links are more comprehensively explored and appreciated to ensure the appropriate valuation of biodiversity, to understand the mechanisms that underlie biodiversity-interest relationships more fully, and assess the potential generality across systems.

## Materials and Methods

### Image-based online study

To elucidate which components of biodiversity influence interest in simulated natural images, we created an online study. Using images composited from photos of natural rocky shores and organisms, we orthogonally manipulated species (4 vs. 8 species; see Supplementary Table 3 for full species lists) and phyletic richness (1, 2 and 4 phyla; Arthropoda, Cnidaria, Echinodermata and Mollusca). Crossing species and phyletic richness, along with variation in species composition, within species and phyletic richness levels, led to continuous variation in functional diversity (the third facet of biodiversity examined here), and the scene-level properties of colourfulness and hue diversity; these variables were also quantified as potential drivers of interest.

**Figure 3:**
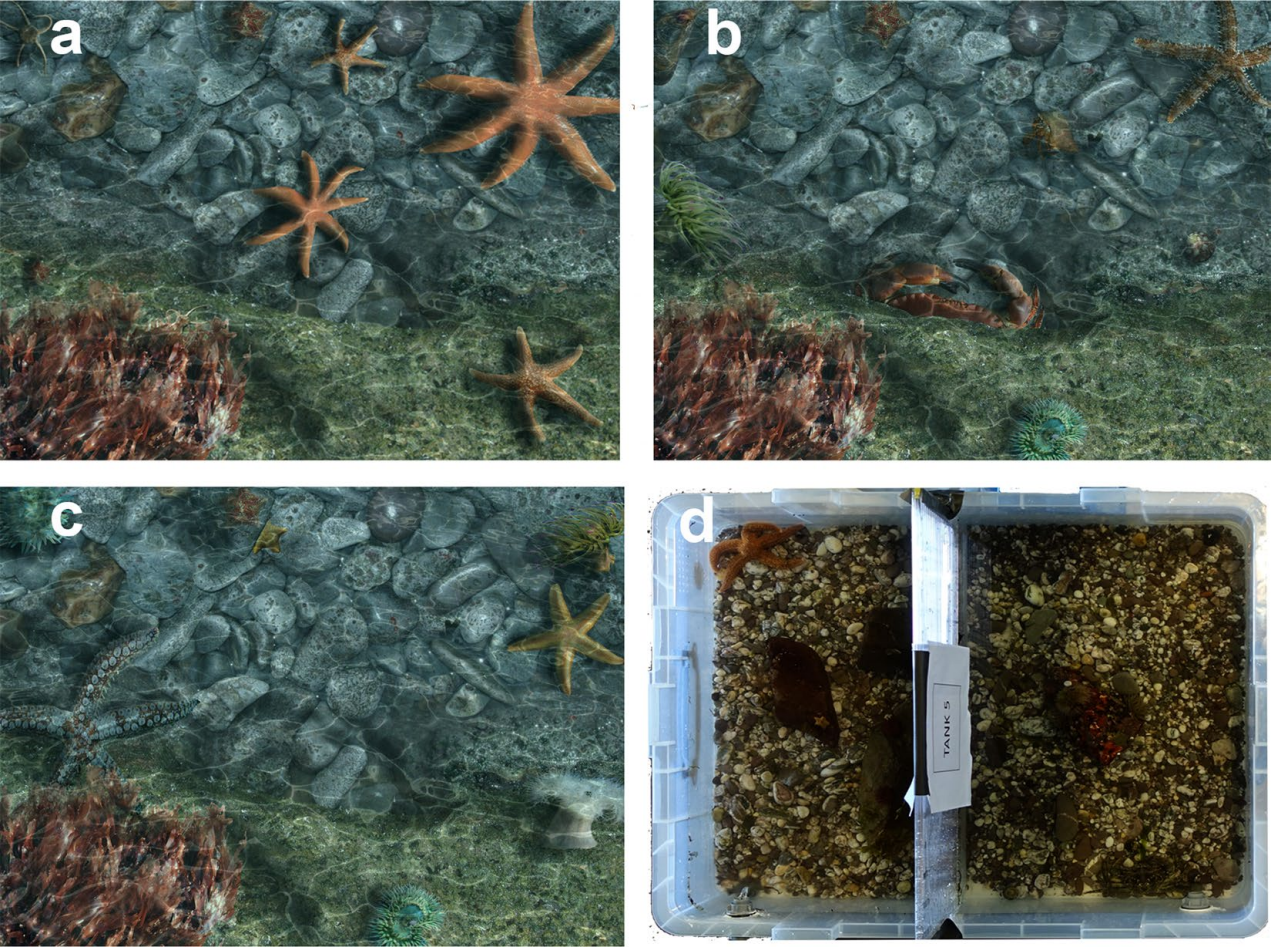
Examples of simulated tide pools for (**a**) Online study (4 Species, 1 phyla), (**b**) Online study (8 Species, 4 phyla), (**c**) Online study (8 Species, 2 phyla) and (**d**) an example tank from the public exhibit study at Wembury Marine Centre.

Creating the images of varying diversity was a three-stage process. First, a diverse set of imaged organisms was compiled. To do this, three different individuals of 32 different animal species (96 individuals) (Supplementary Table 3) from 4 common invertebrate phyla found in tide pools in south Wales (Arthropoda, Cnidaria, Echinodermata and Mollusca) were located in natural tide pools in the field. These individuals were photographed using a Sony® RX100IV camera and Ikelite® housing, before being digitally extracted from their surrounding substrates using Adobe® Photoshop® CC® (2016). Second, background tide pool scenes were compiled. To do this, three images of natural tide pool substrate were taken, as well as additional background images of the common seaweed *Palmaria palmata*, larger pebbles and rocks. Third, images were then composited by setting the images of the animals within pool backgrounds, including seaweed and rocks to create simulated tide pool images (Fig. 3a–c).

In these images, taxonomic richness levels were orthogonally manipulated, creating images containing either 4 or 8 species, selected from 1, 2 or 4 different phyla. Care was taken to ensure that phyletic composition was systematically varied so that all possible phylum combinations were considered within any given level of phyletic richness. This resulted in 11 possible combinations (individual phyla, phyla pairs, all phyla) within each species richness level (4 and 8), giving a total of 22 treatments. Within each of these treatments, species within each phylum were chosen at random. Treatments were replicated by setting them within three slightly varied background scenes to reduce viewer fatigue, yielding a grand total of 66 unique animal-containing images, and three control scenes which had no animals. Across images, the number of animals was held constant (8) and the total image area occupied by animals varied minimally (*M* = 11 ± 0.5% S.D.).

The online study was created and delivered using the SurveyMonkey® online platform. Participants were recruited using social media posts and promotions which linked to the survey, and were shared with people from a wide demography. Participants were informed that the purpose of the experiment was to “understand how the public perceive natural coastal tide pool environments and the animals that live there”, but the specific aims were not stated explicitly to prevent bias from respondents. Participants were asked whether they consented to being included in the study before they progressed, and informed that they were free to withdraw, without penalty, at any time. Participants were presented nine different tide pool images, with the one and four phyla treatments presented once, and two phyla treatments presented twice as there was a greater number of possible phylum combinations in this treatment. Images within each species and phyletic richness treatment were drawn randomly using multi-way A/B split testing in SurveyMonkey®, and then the order of treatments randomized by randomizing page order to avoid order bias.

For each image, participants were asked to rate how “interesting” they found images using a visual-analogue slider which was anchored between “Not at all interesting” (0) and “Extremely interesting” (1), with a midpoint of “Moderately interesting”. Using a slider instead of a typical 5- or 7-point Likert-type item allowed for greater granularity in individual responses while being both comparable to the Likert-type scale used for the public exhibit study^89–91^, and more engaging to participants^91^.

As well as species richness and phyletic diversity, which were directly manipulated, functional diversity of communities varied freely and was measured for each image. Specifically, functional divergence, which captures deviance of species’ traits from the community centroid, was measured using the “FD” package in R^45^. This metric was selected as the divergence of traits in a community was expected to lead to greater perceptible visual differences than species richness, and functional richness (total multidimensional space occupied) could not be calculated where communities had fewer than 3 functionally distinct individuals, with some monophyletic communities containing only 2 functionally distinct entities. Traits, such as body size, type of locomotion and feeding methods, were selected that were linked to the ecology of the animals and were comparable across taxonomic groups (Supplementary Table 4). These were quantified for each individual within an image to allow for intraspecies differences in size to be expressed, and a multidimensional functional space was constructed for community traits using a Gower dissimilarity matrix, in the “Cluster” R package^92^.

For images used in the online study, two whole-image colour-based aesthetic features were also quantified. Colourfulness and the diversity of hues in an image have been found to affect aesthetic preference in previous studies^48,93^, and may mediate the effects of diversity on interest. “Colourfulness” measures saturation-based colourfulness of the whole image-space (including both background and animal contributions) based on psychophysical category scaling^94^. It was calculated using the “getColourfulness.m” function in Matlab 2016 (available at: https://gist.github.com/zabela/8539116.js). Hue diversity measures the diversity of base hues from whole images in HSV (Hue, Saturation, Value) colour space, holding saturation and value constant. Hues were mapped to images using 64 8-bit colour samples taken for uniform colour space (sRGB IEC 61966-2-1:1999) using Adobe® Photoshop® CC® (2016). Images were then transferred to ImageJ and histogram mapping of the reduced colour space undertaken to generate a count of the number of pixels within each hue value, which was saved as a vector. An index of diversity of the colours was then created using Shannon’s diversity index^95^.

Demographic information on gender, age, education level, affiliation with any natural science discipline, and affiliation with a marine biology related discipline were taken for participants to examine demographic influences in interest or perception of diversity. No personally identifiable information was obtained. The study was conducted in adherence to the ethical policies of Swansea University, and the guidelines set out by the British Psychological society. Ethical approval was granted for this experiment by Swansea University College of Science Ethics Board (COS051016-TF).

### Public exhibit study

The public exhibit study was done at Wembury Marine Centre, Devon, UK, from the 14^th^ to 17^th^ of August 2016, to examine how biodiversity affects interest under conditions more representative of the activity of tidepooling. This experiment included the same four phyla as used in the online study but focused on phyletic (not species) richness and if/how its effects were mediated by functional diversity (divergence). Seawater tank exhibits containing different diversity treatments of live tide pool animals were set up with participants, drawn from centre visitors, asked to rate how interesting they found each tank.

The six tank units were deployed in each of six, three hour sessions, either in the morning (10:00–13:00) or afternoon (13:00–16:00), spread across four consecutive days, giving a total of 36 replicate animal assemblages across the experiment, distributed equally among morning and afternoon sessions. On two of the four days, the marine centre had other activities that were run, one during a morning session, and one during an afternoon, and as such only one session was run during each of these days. Within each session, each tank unit was randomly assigned a treatment, consisting of one, two or four phyla, with species richness held constant at four. We also included a control tank with no animals. Each treatment, including the control, was replicated a total of three times, with the four species in each replicate randomly drawn from a pool of 16 species spread evenly among the four focal phyla and available locally (Supplementary Table 5). Functional diversity was not explicitly controlled in the experiment, varying through differences between phyla and species, and within species through representation of different phenotypes (differences in size), and was quantified in the same way as the online study.

Participants were drawn from visitors to Wembury and the Wembury Marine Centre, and were recruited through both posters at the venue, and direct contact with the research assistants where they were asked if they would like to volunteer or not. Each participant was informed that the purpose of the study was to understand how people view nature, informed that they were free to withdraw at any time, and consented to participate, prior to viewing tanks. Where participants were minors, Parental/Guardian consent was also obtained prior to being included in the study. Participants told the experiment was “to examine how different people see different animals” to avoid biasing. Participants were asked to rate each tank on how interesting they found it on a 7-point Likert-type scale, ranging from “not at all interesting” (1) to “Extremely interesting” (7). We also asked for basic demographics (gender, age). Participants were thanked for their participation then debriefed. No personally identifiable information was taken, and the study was carried out in accordance with Swansea Universities’ and the British Psychological Societies’ ethical guidelines. The public exhibit study was granted ethical approval by the Swansea University Human Sciences Ethics Board (HS082016).

Seawater tank exhibits (Fig. 3d) were set-up as follows. First, three 80 L tanks of 1 m × 0.5 m × 0.2 m (length, width, depth) were divided into 2 units using a frosted Perspex divider, yielding 6 tank units. We then covered the bottom of each unit with a thin (1 cm) layer of gravel from the local beach at Wembury and added one large complex stone (~600 cm^3^) to provide some refuge while not completely obscuring organisms from view. Tanks were recirculating, and received seawater pumped (using Eheim Compact 1000 pumps) from attached 30 L plastic sumps containing filtration systems and aeration stones. Each morning half of the total water in the system (55 L) was replaced with fresh seawater from the local shore. Gravity-only returns to the sump maintained water levels in tanks and ensured adequate circulation of water.

### Statistical analysis

Analysis was performed in the statistical computing program R [3.3.2]^96^. The effects of different diversity (species, phyletic, functional (factors)) and colour components (colourfulness, hue diversity (covariates)) on interest were assessed by mixed-effects beta regressions, with each respondent as a random factor (random intercept, fixed slope) to account for variability in baseline interest of participants for the tide pool images, using the glmmADMB package^97^. To constrain interest measures to avoid extremes (0 or 1) which bias estimates, interest was corrected using the equation proposed by Smithson & Verkuilen^98^:

$$y^{\prime\prime} =[y^{\prime} ({\rm{N}}{\textstyle \text{-}}1)+1/2]/{\rm{N}},$$

where *y*″ is the interest index value (0–1), *y*″ is the corrected estimate of *y*′, and N is the number of observations. To parse the direct and indirect effects of diversity components, sets of models were combined within a piecewise Structural Equation Modeling (SEM) (package: “piecewiseSEM”^99^) framework. Justification for model pathways are provided in Supplementary Table 6.

The following two SEM models were created (Supplementary Table 7). The first model used the full data set minus control images (no animals) from the online study (n = 4795 observations). The control images were excluded to avoid confounding the presence/absence of animals with the effect of animal diversity. This model consisted of the direct effects of diversity and colour components on interest (sub-model 1), the direct effects of diversity on functional diversity (sub-model 2), and the direct effects of diversity components on colourfulness (sub-model 3) and hue diversity (sub-model 4). It therefore included pathways of indirect effects from taxonomic diversity components through functional diversity and colour components, and on to interest.

The second SEM model used the full data set minus controls for the public exhibit, with the same rationale for excluding the controls. This model consisted of the direct effects of phyletic richness and functional diversity on interest (sub-model 1), and the direct effect of phyletic diversity on functional diversity, and therefore an indirect effect pathway from phyletic richness to functional diversity and on to interest.

Estimates from the exhibit study, were also used to visualise the partial effects of all hypothesized predictors on interest using the package “coefplot”^100^. An additional glmmADMB regression model was constructed for the online and exhibit studies, including controls, to examine how the presence of animals influenced interest, above the inherent level of interest people have in tide pool images devoid of animals. Both species richness and phyletic richness were treated as continuous variables, and all predictors were scaled, creating z-scores.

In both online and public exhibit studies, the effect of phyletic identity was also examined to determine if the presence of a particular phylum drove interest, or whether diversity effects were the most important in determining interest (see Isbell *et al*.^55^ for a similar approach). Binary presence/absence (1/0) scores were coded as factors and analysed in glmmADMB using a beta error family, with respondent as a random factor. The effects of phyla were visually represented using “coefplot”.

For both the online and exhibit studies we analysed the effect of participants’ demographics on interest using similar glmmADMB model structures described above. We treated education level, affiliation with any natural science discipline, affiliation with a marine biology related discipline (online only), gender and age range (exhibit and online studies) as additive factors in regression models. To collapse factors, a mixed effects Anova was performed on the glmmADMB models using the “CAR” package^101^.

## Electronic supplementary material

Supplementary Information

## Data Availability

The datasets analysed during this study are available from the data sharing service Figshare (10.6084/m9.figshare.7072091.v1).
