# Supplementary material for: Multiple dimensions of biodiversity drive human interest in tide pool communities
Source: Sci Rep. 2018 Oct 15;8:15234. doi: 10.1038/s41598-018-33155-x (PMC6189033; doi:10.1038/s41598-018-33155-x)
Supplement: Supplementary file 1 — Supplementary Information [file 41598_2018_33155_MOESM1_ESM.pdf]

Supplementary Information

Multiple dimensions of biodiversity drive human interest in tide pool communities

Tom P. Fairchild*[a], Mike S. Fowler[a], Sabine Pahl[b], John N. Griffin[a]

[a]Department of Biosciences, Swansea University, Singleton Park Campus, Swansea, SA2 8PP, UK

[b]School of Psychology, Plymouth University, 22 Portland Square, Drake Circus, Plymouth, Devon, PL4 8AA.

*Corresponding author

tom.phillip.fairchild@googlemail.com; Tel: 07828315263

Supplementary Table 1: Of the 741 people that responded to the main questionnaire, 601 completed the image-based pool scene questions, and 527 people completed the questionnaire and demographics in full, giving a completion rate of 71.1%. Of those that answered the demographic questions, 315 (59.8%) identified as being female, 207 (39.7%) as male and 2 as being an alternative gender (0.3%). 3 people preferred not to state their gender. People within the 18-30 age group were the most represented (n=368, 69.8%) and under 18s (n=5, 1%) and over 70s (n=7, 1.3%) the least represented. Respondents with degree-level or above (degree; n=134, postgraduate-degree: n=176) qualifications constituted the largest group at 59% (n=310). Those with A-levels or equivalent as their highest qualification were also well represented (n=180, 32.2%), with few respondents stating their highest qualification at a lower level, or no qualifications (n= 24, 4.6%). While those who work or study in natural sciences were over-represented (n=324, 61.5%), most participants did not work or study in a marine biology related field (n=310, 58.8%). The mixed effect Anova table (Type-II Sums of Squares) below shows the effect of demographic background on interest for. Only achromatopsia colour-blindness affected interest, but had a very small sample size (n=5) and as such was disregarded.

Demographic Category	Degrees of Freedom	Chi squared	P value
Gender	1	0.1659	0.683
Age	4	2.4867	0.647
Academic Qualifications	4	7.4307	0.114
Environmental Science	4	1.0961	0.895
Colourblindness	3	11.2466	0.010

Supplementary Table 2: 109 people took part in the public exhibit study, of which 97 provided demographic information. The median age group of respondents was 31-50 (n=34), with children (0-10) also making up a large portion of the demographic (n=28), with the remaining participants split between 11-18 age group (n=15), 51-70 age group (n=13), 19-30 age group (n=4), and 70+ age group (n=3). Participants were 33% males (n=33), and 67% females (n=66). Neither age, nor gender had any significant effect (α=0.05) on interest. The mixed effect Anova table (Type-II Sums of Squares) below shows the effect of demographic background on interest.

Demographic Category	Degrees of Freedom	Chi squared	P value
Gender	1	1.389	0.239
Age	5	10.368	0.066

Supplementary Table 3: Species and phyla used in the online study images.

Species	Phylum
Pilumnus hirtellus<br>*Carcinus maenas*<br>*Cancer pagurus*<br>*Pagurus bernhardus*<br>*Palaemon serratus*<br>*Necora puber*<br>*Liocarcinus depurator*<br>*Galathea squamifera*	Arthropoda
Actinia equina<br>*Actinia fragacea*<br>*Actinothoe sphyrodeta*<br>*Anemonia viridis*<br>*Urticina felina*<br>*Sagartia elegans*<br>*Metridium senile*<br>*Lucernariopsis campanulata*	Cnidaria
Amphipholis squamata<br>*Asterias rubens*<br>*Asterias gibbosa*<br>*Henricia oculata*<br>*Marthasterias glacialis*<br>*Psammechinus miliaris*<br>*Luidia ciliaris*<br>*Asterina phylactica*	Echinodermata
Leptochiton scabridus<br>*Mytilus edulis*<br>*Phorcus linearis*<br>*Patella vulgata*<br>*Gibbula umbilicalis*<br>*Nucella lapilus*<br>*Littorina saxatilis*<br>*Patella depressa*	Mollusca

Supplementary Table 4: Trait types and measures used to construct Gower dissimilarities for functional diversity metrics. Measurements for size differed between studies, with continuous area measurements used for the online study, and size factors (small, medium and large) for the public exhibit study.

Trait type	Measure	Type
Size	Surface area of animal	Continuous (cm^2) [ONLINE], Factor (Small, Medium, Large) [PUBLIC EXHIBIT]
Body form	Presence of Exoskeleton	Binary
Locomotion	Swimming species	Binary
	Walking species	Binary
	Wave-locomotion via muscular foot species	Binary
	Locomotive ability	Ordered factor; (0) sessile, (1) limited mobility, mostly immobile, (2) Mobile, slow moving, (3) Mobile, fast moving.
	Does the species have obvious walking appendages?	Binary
Trophic niche	Trophic Level	Ordered factor 1-3, From "Biotic"
	Suspension feeding species	Binary
	Active predatory species	Binary
	Scavenging species	Binary
	Grazing species	Binary
Aesthetics	Dominant organism colour	Factor; "White", "Brown", "Red", "Yellow", "Green", "Blue", "Purple", "Black"

Supplementary Table 5: Species and phyla used in the public exhibit study images.

Species	Phylum
Carcinus maenas	Arthropoda
Cancer pagurus	
Pagurus bernhardus	
Necora puber	
Actinia equina	Cnidaria
Actinia fragacea	
Anemonia viridis	
Urticina felina	
Asterias rubens	Echinodermata
Asterias gibbosa	
Marthasterias glacialis	
Psammechinus miliaris	
Phorcus linearis	Mollusca
Patella vulgata	
Gibbula umbilicalis	
Nucella lapilus	

Supplementary Table 6: Justifications for each pathway contained in the online and public exhibit studies. References are presented at the end of the supplementary document.

Response	Predictor	Justification(references)
Interest	Species Richness	Species richness is the most common biodiversity metric, and has been found to be an important component affecting preference in previous studies[1,2].
Interest	Phyletic Richness	Captures higher level of taxonomic diversity and divergence in traits, increasing visual complexity which may be more perceptible to lay observers and may increase interest[3].
Interest	Functional Diversity	Functional diversity metrics increase preference for scenes by increasing the perceivable visual complexity of communities within an image[2,3].
Interest	Colourfulness	The colourfulness, and related measures of saturation, of images have been strongly linked to preference for natural scenes and photographs[2,4].
Interest	Hue diversity	Diversity of hues expressed within a scene add additional visually distinct complexity, and where diversity is high are generally preferred in natural scenes[5,6].
Functional Diversity	Species Richness	Species richness positively influences functional diversity by increasing the probability of capturing functionally different species[7,8].
Functional Diversity	Phyletic Richness	Evolutionary distance is greater between phyla than within phyla, with evolution, and differentiation, of traits likely to be greater in communities that have greater evolutionary separation[9].
Colourfulness	Species Richness/ Phyletic Richness	It was expected that increasing the number of species in a community would increase the probability of including more colourful species/phyla and a greater variety of hues[10].
Colourfulness/hue diversity	Functional Diversity	Dominant colour of organisms was included as a trait and it was expected that colour of individual organisms would influence overall scene colourfulness and hue diversity.<br>Increasing functional diversity was likely to increase the probability of including species which were more colourful or diverse[10].

Supplementary Table 7: Model specification for SEM models, including glmmADMB sub-model responses and predictors.

SEM Model	Sub-model	Response	Predictors
1 Online (full)	1	Interest	Species richness, Functional diversity, Phyletic richness, Colourfulness, Hue diversity
	2	Functional Diversity	Species richness, Phyletic richness
	3	Colourfulness	Species richness, Functional diversity, Phyletic richness,
	4	Hue diversity	Species richness, Functional diversity, Phyletic richness
2 Public Exhibit	1	Interest	Phyletic richness, Functional diversity
	2	Functional diversity	Phyletic richness

Supplementary References

1. Lindemann-Matthies, P., Junge, X. & Matthies, D. influence of plant diversity on people's perception and aesthetic appreciation of grassland vegetation. *Biol. Conserv.* (2010).

2. Tribot, A. S. *et al.* Taxonomic and functional diversity increase the aesthetic value of coralligenous reefs. *Sci. Rep.* **6,** (2016).

3. Ulrich, R. S. Biophilia, biophobia, and natural landscapes. *Biophilia Hypothesis* **7,** 73–137 (1993).

4. Simonič, T. Preference and perceived naturalness in visual perception of naturalistic landscapes. *J. Biotechnol.* **81,** 369–38 (2003).

5. Fortmann-Roe, S. Effects of hue, saturation, and brightness on color preference in social networks: Gender-based color preference on the social networking site Twitter. *Color Res. Appl.* **38,** 196–202 (2013).

6. Linhares, J. M. M. *et al.* Viewing natural scenes through colored filters. *Invest. Ophthalmol. Vis. Sci.* **46,** 4686–4686 (2005).

7. Petchey, O. L. & Gaston, K. J. Functional diversity (FD), species richness and community composition. *Ecol. Lett.* **5,** 402–411 (2002).

8. Cadotte, M. W., Carscadden, K. & Mirotchnick, N. Beyond species: functional diversity and the maintenance of ecological processes and services. *J. Appl. Ecol.* **48,** 1079–1087 (2011).

9. Martiny, A. C., Treseder, K. & Pusch, G. Phylogenetic conservatism of functional traits in microorganisms. *ISME J.* **7,** 830–838 (2013).

10. Huston, M. A. Hidden treatments in ecological experiments: re-evaluating the ecosystem function of biodiversity. *Oecologia* **110,** 449–460 (1997).
